# Elevated Serum Protease 3 Antineutrophil Cytoplasmic Antibody in Mesalazine-Intolerant Ulcerative Colitis: A Potential Diagnostic Biomarker

**DOI:** 10.3390/jcm14197019

**Published:** 2025-10-03

**Authors:** Yuhei Oyama, Takashi Taida, Yoshiki Matsubara, Tomomi Ozaki, Takuya Ohashi, Toshiyuki Ito, Shohei Mukai, Nobuaki Shu, Yushi Koshibu, Yusuke Ozeki, Makoto Furuya, Yukiyo Mamiya, Hayato Nakazawa, Ryosuke Horio, Chihiro Goto, Satsuki Takahashi, Akane Kurosugi, Michiko Sonoda, Tatsuya Kaneko, Tsubasa Ishikawa, Yuki Ohta, Kenichiro Okimoto, Keiko Saito, Tomoaki Matsumura, Jun Kato

**Affiliations:** 1 Department of Gastroenterology, Graduate School of Medicine, Chiba University, Chiba 260-8677, Japan; oyama.yuhei@chiba-u.jp (Y.O.); taida@chiba-u.jp (T.T.); matsubaray@chiba-u.jp (Y.M.); ozaki.tomomi@chiba-u.jp (T.O.); ohashit@chiba-u.jp (T.O.); ito.t@chiba-u.jp (T.I.); mukai.shohei@chiba-u.jp (S.M.); shu.nobuaki@chiba-u.jp (N.S.); koshibu.yushi@chiba-u.jp (Y.K.); ozeki.yusuke@chiba-u.jp (Y.O.); furuya-makoto@chiba-u.jp (M.F.); fukumura-yukiyo@chiba-u.jp (Y.M.); nakazawa.hayato@chiba-u.jp (H.N.); horio-ryosuke@chiba-u.jp (R.H.); goto-chihiro@chiba-u.jp (C.G.); takahashi-satsuki@chiba-u.jp (S.T.); akanekurosugi1125@chiba-u.jp (A.K.); sonoda.michi@chiba-u.jp (M.S.); kanekota@chiba-u.jp (T.K.); ishikawa.tsubasa@chiba-u.jp (T.I.); ohta.yuki@chiba-u.jp (Y.O.); okimoto-k@chiba-u.jp (K.O.); keiko-ueda@chiba-u.jp (K.S.); matsumura@chiba-u.jp (T.M.); 2 Endoscopy Center, Chiba University Hospital, Chiba 260-8677, Japan

**Keywords:** ulcerative colitis, mesalazine intolerance, proteinase 3 antineutrophil cytoplasmic antibody

## Abstract

**Background/Objectives**: Mesalazine agents are essential drugs for treating ulcerative colitis (UC). Biomarkers that can differentiate mesalazine intolerance from exacerbated UC are needed because of the similarity of their symptoms and increasing prevalence of mesalazine intolerance. The study aim was to assess the usefulness of proteinase 3 antineutrophil cytoplasmic antibody (PR3-ANCA) to identify mesalazine intolerance in patients with UC. **Methods**: In this single-center retrospective study, patients with UC in whom serum PR3-ANCA was measured were included, and the serum levels were compared between the mesalazine-intolerant and -tolerant patient groups. The predictability of the marker to discriminate between these patients was analyzed. **Results**: Among 406 patients with UC with measured serum PR3-ANCA levels, 68 (17%) had mesalazine intolerance. The PR3-ANCA levels were significantly higher in the intolerance group than in the tolerance group [4.5 U/mL (0.8–26.2 U/mL) vs. 1.5 U/mL (0.0–8.5 U/mL), *p* = 0.001]. The area under the curve of the receiver operating characteristic curve analysis of the predictability of PR3-ANCA in differentiating mesalazine-intolerant patients from clinically active patients with UC was 0.755 (95% confidence interval: 0.634–0.876, cutoff value: 15.05 U/mL; sensitivity: 0.625, specificity: 0.813). Multivariate logistic regression analysis using various clinical factors revealed that serum PR3-ANCA > 15.0 U/mL was an independent risk factor of mesalazine intolerance (odds ratio: 8.25, 95% confidence interval: 2.52–27.02, *p* < 0.001). **Conclusions**: Serum PR3-ANCA could be a useful marker to identify mesalazine-intolerant patients with UC.

## 1. Introduction

Ulcerative colitis (UC) is an idiopathic and chronic inflammatory disease of the colonic mucosa. It is characterized by bloody stools and diarrhea as its primary symptoms, with repeated remissions and relapses [1]. The number of patients with UC is still increasing worldwide, and no cure has been established. Therefore, UC has become one of the most common diseases to be treated by gastroenterologist [2]. Although the emergence of various biological and small-molecule agents has evolved the management of UC, mesalazine agents remain the essential therapy for most patients [3,4].

Mesalazine agents are important not only for the induction of remission but also for long-term maintenance of UC remission and are considered very safe [5,6]. Recently, however, mesalazine intolerance has been increasingly observed in patients in UC [7] and has become a serious problem in UC management. The symptoms of mesalazine intolerance include fever, abdominal pain, and bloody stools, which are similar to those of exacerbation of UC [8], so differentiating mesalazine intolerance from UC exacerbation clinically is sometimes difficult. For patients with mesalazine intolerance, any further medication is ineffective unless mesalazine is discontinued. Therefore, biomarkers that discriminate mesalazine intolerance from UC exacerbation are urgently needed.

Although several biomarkers are associated with the disease activity of UC, including fecal calprotectin [9], fecal occult blood [10,11], and recently reported leucine-rich glycoprotein [12], none have proven helpful in discriminating mesalazine intolerance. The drug-induced lymphocyte stimulation test (DLST) has been occasionally assessed in patients suspected of mesalazine intolerance, but its ability to identify mesalazine intolerance is controversial [13].

Antineutrophil cytoplasmic antibody (ANCA), a marker of systemic vasculitis, has long been considered a useful biomarker of UC. Elevated serum proteinase 3 antineutrophil cytoplasmic antibody (PR3-ANCA) levels, which is the quantity of the antibodies to proteinase 3 (PR3), diffusely present in the cytoplasm of neutrophils, is helpful for discriminating patients with UC from those without [14,15]. In addition, patients with UC with elevated serum PR3-ANCA levels are more likely to not respond well to steroid therapy [16] and to encounter primary nonresponse to antitumor necrosis factor-α agents [17]. A previous case report described a patient with mesalazine intolerance who had an elevated serum PR3-ANCA level [18].

Considering these reports, PR3-ANCA-positive cases appear to represent a distinct subpopulation of UC that shows phenotypes of treatment refractoriness, including mesalazine intolerance. However, no studies have reported the relationship between mesalazine intolerance and PR3-ANCA. The current study aimed to investigate the utility of serum PR3-ANCA as a biomarker of mesalazine intolerance in UC.

## 2. Materials and Methods

### 2.1. Patients

This retrospective study included consecutive patients with a confirmed diagnosis of ulcerative colitis (UC) who were treated at Chiba University Hospital between January 2020 and December 2023. UC diagnosis was made based on a combination of clinical, endoscopic, and histological findings, in accordance with internationally accepted guidelines [19,20,21].

Patients were eligible for this study if they had a confirmed diagnosis of UC and had their serum PR3-ANCA levels measured at their first visit to our hospital. Patients were excluded if they had a diagnosis of ANCA-associated vasculitis or a history of total or subtotal colectomy prior to PR3-ANCA measurement.

The following information was collected and analyzed when the serum PR3-ANCA level was measured at the first visit to the hospital: sex, age, disease duration, disease extent, extraintestinal manifestations, medications, clinical symptoms, and blood test results, including PR3-ANCA and C-reactive protein (CRP). The partial Mayo (pMayo) score, a disease activity index in which stool frequency, rectal bleeding, and the physician’s global assessment are each scored on a scale from 0 to 3, resulting in a total score ranging from 0 to 9 [22], were used to assess clinical symptoms.

The following detailed information regarding the administration of mesalazine agents was obtained from each patient’s start of administration until December 2023: brand names, start and discontinuation timepoints (day), administered dose, dose increase and dose reduction, and adverse events, including mesalazine intolerance.

### 2.2. Definition of Mesalazine Intolerance

According to the criteria specified in several studies [23,24], we defined mesalazine intolerance as an apparent worsening of symptoms, such as fever, diarrhea, bloody stool, or abdominal pain, after starting or increasing the dose of a mesalazine agent, which improved immediately after the medication was discontinued. PR3-ANCA measurements were performed retrospectively as an adjunctive diagnostic tool or disease assessment in UC; therefore, clinicians were not aware of the relation between PR3-ANCA and mesalazine intolerance at the time of diagnosis.

### 2.3. Measurement of PR3-ANCA

Serum PR3-ANCA levels were measured using blood samples collected at the time of the patient’s first visit to our hospital. The serum PR3-ANCA level was measured by an external laboratory using the STACIA^®^ MEBLux™ Test kit (Medical & Biological Laboratories Co., Ltd., Tokyo, Japan), which uses a chemiluminescent enzyme immunoassay; the lower limit of detection for PR3-ANCA is 1.0 U/mL, and the cutoff value for healthy adults is set at 3.5 U/mL. At our institution, this testing method has been consistently adopted since 2013. This method is universally available in clinical practice. Cases with the measured serum PR3-ANCA less than the detection limit were analyzed as 0.0 U/mL.

### 2.4. Study Endpoint

The primary endpoint was to assess the possible usefulness of serum PR3-ANCA in identifying mesalazine intolerance among patients with UC. The secondary endpoint was to demonstrate the predictability of PR3-ANCA for mesalazine intolerance using a cutoff value.

### 2.5. Statistical Analysis

Study group analyses were performed using the Shapiro–Wilk test, Mann–Whitney test, χ^2^ test, and Fisher’s exact probability test, as appropriate. Continuous variables are described by the median (interquartile range [IQR]). The cutoff value for serum PR3-ANCA was determined by creating a receiver operating characteristic (ROC) curve using the Youden index. To identify independent risk factors for mesalazine intolerance, a multivariate logistic regression analysis was applied that used sex, age, age at diagnosis, disease duration, disease extent, extraintestinal manifestations, medications (including systematic corticosteroids, topical corticosteroids, thiopurines, calcineurin inhibitors, biologics (Bio)/Janus kinase (JAK) inhibitors and others), pMayo score, serum CRP levels, and serum PR3-ANCA levels as confounding factors. Factors with values of *p* < 0.05 in the univariate analysis were included in the multivariate analysis. Statistical significance was defined as values of *p* < 0.05. IBM SPSS Statistics version 26 (IBM Corp., Armonk, NY, USA) was used for statistical analysis and figure preparation. In this study, no generative artificial intelligence (GenAI) has been used.

## 3. Results

### 3.1. Study Patients

**Figure 1:**
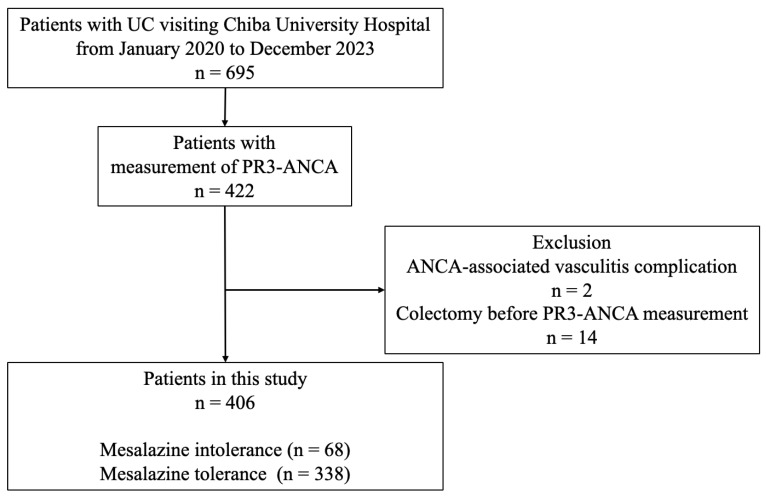
Study flow diagram. Of the 406 patients with UC with measured PR3-ANCA levels, 68 (17%) were classified into the mesalazine intolerance group, and 338 (83%) were classified into the mesalazine tolerance group. ANCA: antineutrophil cytoplasmic antibody; PR3-ANCA: protease 3 antineutrophil cytoplasmic antibody; UC: ulcerative colitis.

A total of 695 patients with UC visited Chiba University Hospital between January 2020 and December 2023. Of these, 422 patients had their serum PR3-ANCA levels measured at their first visit since January 2013. After excluding two patients with ANCA-associated vasculitis and 14 patients who had undergone colectomy before the measurement of serum PR3-ANCA levels, 406 patients were included in this study. Mesalazine intolerance was observed in 68 (17%) patients, whereas the remaining 338 (83%) patients were considered mesalazine-tolerant (Figure 1).

The characteristics of the 406 study patients, with comparison between the mesalazine intolerance and mesalazine tolerance group, are shown in Table 1. The median disease duration was 2.5 years (0.0–10.0 years), and the median serum CRP and PR3-ANCA levels were 0.16 mg/dL (0.04–1.17 mg/dL) and 1.9 U/mL (0.0–10.5 U/mL), respectively. The intolerance group had a significantly shorter disease duration [1.0 years (0.0–7.3 years) vs. 3.0 years (0.0–11.0 years), *p* = 0.027], fewer patients taking oral mesalazine agents [30 (44%) vs. 242 (72%), *p* < 0.001], higher serum CRP levels [0.42 mg/dL (0.08–5.03 md/dL) vs. 0.13 mg/dL (0.03–0.87 mg/dL), *p* = 0.003], and higher PR3-ANCA levels [4.5 U/mL (0.8–26.2 U/mL) vs. 1.5 U/mL (0.0–8.5 U/mL), *p* = 0.001] than the tolerance group, but the other characteristics were not significantly different between the two groups.

### 3.2. Timings of the Measurement of Serum PR3-ANCA Level and Appearance of Symptoms of Mesalazine Intolerance

Because the timing of measurement of serum PR3-ANCA did not always coincide with the appearance of symptoms of mesalazine intolerance in each patient, the results of serum PR3-ANCA were shown on the basis of the timing of the appearance of symptoms of intolerance (Table 2). The highest PR3-ANCA value was 17.3 U/mL (3.4–49.7 U/mL) when measured during the appearance of symptoms. However, the values in patients measured before the onset of intolerance symptoms were 4.6 U/mL (0.4–40.1 U/mL), which were still higher than those in patients with mesalazine tolerance. In patients measured after the onset of symptoms, the PR3-ANCA values tended to decrease as the time between the onset and measurement increased.

### 3.3. Serum PR3-ANCA Levels in Clinically Active Patients Between the Mesalazine Intolerance and Tolerance Groups

According to the above results, serum PR3-ANCA levels were highest at the appearance of intolerant symptoms, and discrimination of intolerant symptoms from clinically active UC symptoms is the most relevant in clinical practice. Therefore, the analyses regarding the predictability of PR3-ANCA for mesalazine intolerance were performed in patients with active disease at the measurement of PR3-ANCA (pMayo score ≥ 3), including 16 mesalazine-intolerant patients with measurement of PR3-ANCA during the appearance of intolerant symptoms as cases and 139 mesalazine-tolerant patients as controls.

**Figure 2:**
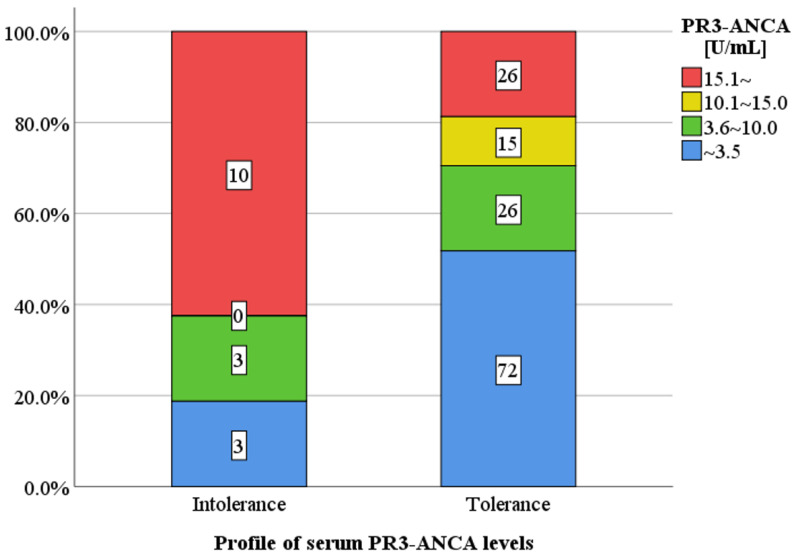
Serum PR3-ANCA levels in clinically active patients between the mesalazine. intolerance and tolerance groups. The proportion of patients with a markedly higher PR3-ANCA level (≥15.1 U/mL) was significantly higher in the intolerance group than in the tolerance group (63% vs. 19%, *p* < 0.01), but the proportion of patients with a normal PR3-ANCA level (≤3.5 U/mL) was significantly lower in the intolerance group than in the tolerance group (19% vs. 52%, *p* = 0.02). PR3-ANCA: protease 3 antineutrophil cytoplasmic antibody.

The profile of serum PR3-ANCA levels for patients in the mesalazine intolerance and tolerance groups is shown (Figure 2). The proportion of patients with a markedly higher PR3-ANCA level (≥15.1 U/mL) was significantly higher in the intolerance group than in the tolerance group (63% vs. 19%, *p* < 0.001), whereas the proportion of patients with a normal PR3-ANCA level (≤3.5 U/mL) was significantly lower in the intolerance group than in the tolerance group (19% vs. 52%, *p* = 0.02).

### 3.4. Accuracy of PR3-ANCA as a Serum Marker in Mesalazine Intolerance

**Figure 3:**
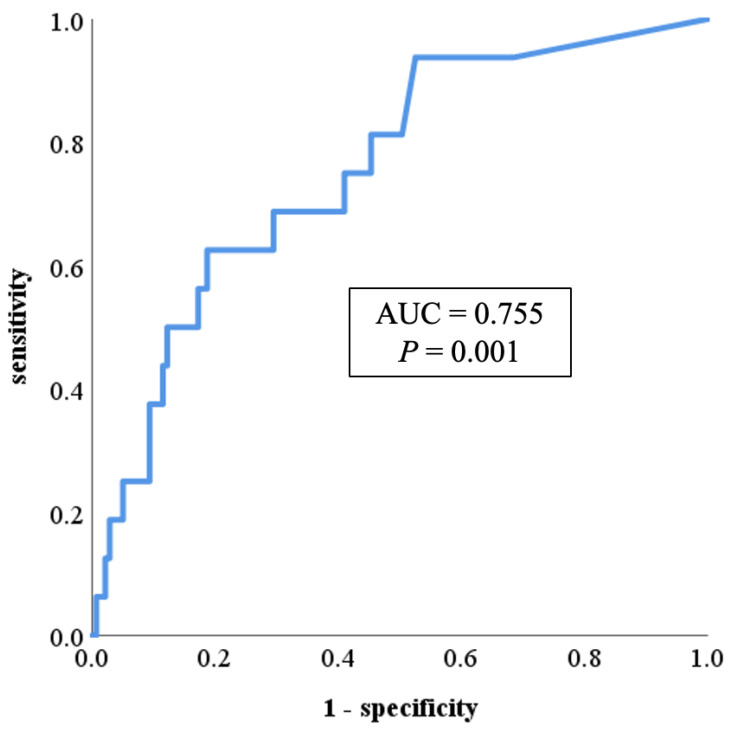
ROC curve showing the relationship between mesalazine intolerance and serum PR3-ANCA levels. The ROC curve analysis showed that the area under the curve was 0.755 (95% CI: 0.634–0.876, *p* = 0.001). The sensitivity and specificity to intolerance were 0.625 and 0.806, respectively, when the cutoff value of serum PR3-ANCA was 15.0 U/mL. AUC: area under the curve; CI: confidence interval; PR3-ANCA: protease 3 antineutrophil cytoplasmic antibody; ROC: receiver operating characteristic.

Using these cases and controls, the predictive potential of PR3-ANCA for mesalazine intolerance was determined by ROC curve analysis. The ROC curve analysis showed that the area under the curve (AUC) was 0.755 [95% confidence interval (CI): 0.634–0.876, *p* = 0.001]. The sensitivity and specificity to intolerance were 0.625 and 0.806, respectively, when the cutoff value of serum PR3-ANCA was 15.0 U/mL (Figure 3).

### 3.5. Analysis of Factors Associated with Mesalazine Intolerance

The results of the multivariate logistic regression analysis using these cases and controls are presented in Table 3. In the univariate analysis, systemic corticosteroids [odds ratio (OR): 3.85, 95% CI: 1.31–11.28, *p* = 0.014], serum CRP levels (OR: 8.17, 95% CI: 1.05–63.69, *p* = 0.045), and serum PR3-ANCA levels (OR: 7.24, 95% CI: 2.42–21.72, *p* < 0.001) were significant risk factors for mesalazine intolerance. In the multivariate analysis, systematic corticosteroids (OR: 3.74, 95% CI: 1.14–12.33, *p* = 0.030) and serum PR3-ANCA levels (OR: 8.25, 95% CI: 2.52–27.02, *p* < 0.001) were identified as independent risk factors for mesalazine intolerance.

## 4. Discussion

Symptoms of mesalazine intolerance often mimic the symptoms of UC exacerbation, and discrimination between them is often difficult clinically [8]. The increasing prevalence of mesalazine intolerance also has a major impact on the clinical practice of UC [7,20]. In the present study, we demonstrated that serum PR3-ANCA level was a useful predictive marker of mesalazine intolerance in patients with UC. To the best of our knowledge, this is the first study to clarify the relationship between mesalazine intolerance and PR3-ANCA. The cutoff value of serum PR3-ANCA for discriminating mesalazine-intolerant patients was markedly higher (>15 U/mL) than the standardized cutoff value (≥3.5 U/mL) that has been used to discriminate between patients with and without UC [14,15]. Therefore, measuring serum PR3-ANCA levels could be helpful not only in the diagnosis of UC but also for discriminating mesalazine intolerance from UC exacerbation.

PR3-ANCA was originally known as a useful diagnostic marker for UC, and Imakiire S. et al. showed that PR3-ANCA ≥ 3.5 U/mL had a sensitivity of 0.445 and a specificity of 0.956 for diagnosing UC [14]. It has also been reported that patients with UC who are PR3-ANCA-positive have higher disease severity and longer disease extension than those who are PR3-ANCA-negative [25]. Furthermore, PR3-ANCA-positive UC is associated with treatment resistance [16], steroid-requirement at diagnosis, and a primary nonresponse to antitumor necrosis factor-α agents [17]. Considering our results, at least part of these refractory disease phenotypes in patients who are PR3-ANCA-positive may be caused by mesalazine intolerance because most patients with UC, even in medication-refractory situations, continue to receive mesalazine. To prove this hypothesis, clinical trials on the discontinuation of mesalazine agents in patients with PR3-ANCA-positive refractory UC are needed.

**Figure 4:**
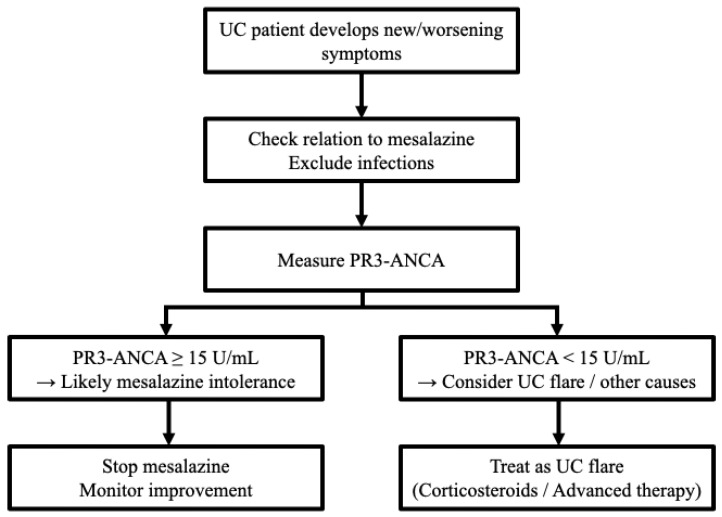
The flowchart regarding a recommended decision-making pathway integrating PR3-ANCA measurement. PR3-ANCA: protease 3 antineutrophil cytoplasmic antibody; UC: ulcerative colitis.

The present study showed that mesalazine intolerance was more common in our patients with shorter disease duration [intolerance group: 1.0 years (0.0–7.3 years) vs. tolerance group: 3.0 years (0.0–11.0 years)]. Similar results have been obtained in previous cohort studies (intolerance group: 0.8 ± 3.0 years vs. tolerance group: 7.4 ± 8.4 years) [26]. These results are unsurprising given that mesalazine is administered in the early stages of UC. According to our results, PR3-ANCA had the best predictive accuracy when measured during mesalazine administration. Therefore, for diagnosing mesalazine intolerance, it is advisable to measure PR3-ANCA at the time of appearance of intolerant symptoms, which often mimic the symptoms of UC exacerbation, at the start of mesalazine administration in the early disease stages to maximize diagnostic yield (Figure 4).

Our results indicated that serum PR3-ANCA was elevated even before the onset of mesalazine intolerance, suggesting that measuring serum PR3-ANCA before administration of mesalazine agents can predict mesalazine intolerance and help physicians avoid mesalazine administration for possible mesalazine-intolerant patients. Once intolerance symptoms occur after the administration of mesalazine to intolerant patients, the prognosis is reportedly poor and includes a high rate of colectomy [27]. Therefore, measurement of serum PR3-ANCA before mesalazine administration could prevent patients from experiencing intolerant symptoms and improve their prognosis. The addition of PR3-ANCA testing to the diagnostic flow may also allow earlier differentiation between mesalazine intolerance and disease flare, potentially reducing unnecessary escalation of immunosuppressive therapy or further investigations. In contrast, in patients measured after symptom onset, the PR3-ANCA values tended to decrease as the time between symptom onset and measurement increased. This result is consistent with the report of Imakiire S. et al. in which PR3-ANCA levels were significantly decreased in patients in clinical remission [14]. For patients who discontinued mesalazine agents because of suspected mesalazine intolerance, serum PR3-ANCA should be measured early after discontinuation for a definitive diagnosis of intolerance.

Several possible inferences regarding the biological mechanisms of serum PR3-ANCA elevation due to mesalazine intolerance can be considered. ANCA antigens are some of the components of neutrophil extracellular traps (NETs) released during neutrophil activation. NETosis, the cell death of neutrophils forming NETs, is involved in the appearance of the autoantibodies [28]. Various drugs, including sulfasalazine and mesalazine, are involved in the development of NETosis [29,30]; therefore, mesalazine-induced NETosis may be correlated with the elevation of PR3-ANCA in patients with mesalazine intolerance. Another possible mechanism involves the inhibition of the nuclear factor-κB (NF-κB) pathway by mesalazine. This inhibition induces neutrophil apoptosis [31], which translocates ANCA antigens to the cell surface and stimulates ANCA production. To assess the usefulness of serum PR3-ANCA levels and to identify more reliable biomarkers for the diagnosis of mesalazine intolerance, further studies regarding the underlying mechanisms are needed.

A reaction to the additives contained in the agents may be another possible mechanism of mesalazine intolerance and PR3-ANCA elevation. Three mesalazine formulations are currently available in Japan: time-dependent mesalazine (Pentasa^®^, Kyorin Pharmaceutical Co., Ltd., Tokyo, Japan), pH-dependent mesalazine (Asacol^®^, Zeria Pharmaceutical Co., Ltd., Tokyo, Japan), and multimatrix system mesalazine (Lialda^®^, Mochida Pharmaceutical Co., Ltd., Tokyo, Japan). These three formulations have different release characteristics in the colon [32,33,34] and, therefore, contain different additives. In our analysis, the prevalence of mesalazine intolerance and serum PR3-ANCA levels in intolerant patients did not differ significantly among these formulations, suggesting that the causes of these phenomena are probably due to mesalazine itself and not to the additives.

DLST has been reported as a method for evaluating allergic reactions to mesalazine agents. Saito D. et al. reported that the sensitivity and specificity of DLST were 0.240 and 0.805 for diagnosing mesalazine intolerance, respectively [13]. In our study, serum PR3-ANCA levels > 15.0 U/mL had a sensitivity of 0.625 and specificity of 0.813 for mesalazine intolerance in patients with active UC, suggesting that it could be a better marker than DLST. Suzuki K. et al. used genetic data combined with clinical data to create a predictive model of mesalazine intolerance that had higher sensitivity (0.714) and specificity (0.908) for predicting mesalazine intolerance [35]. However, genetic testing is very expensive and takes a long time to perform. In contrast, the measurement of PR3-ANCA is commercially available and could be more useful in clinical practice.

A potential limitation of this study is that it was a retrospective analysis conducted at a single center, which can introduce patient selection bias and limited generalizability. In addition, the lack of a clear definition of mesalazine intolerance at present may have led to a somewhat inaccurate retrospective evaluation of mesalazine intolerance. However, mesalazine intolerance was present in 17% of our UC cases, and the prevalence was similar to the results of a multicenter survey conducted in Japan [7]. Therefore, our results of the predictive value of serum PR3-ANCA for mesalazine intolerance will probably be broadly applicable. Future prospective studies with standardized diagnostic criteria are needed to better differentiate these conditions. In addition, the median PR3-ANCA levels were higher in patients with mesalazine intolerance, overlap existed between groups. Since PR3-ANCA has been previously reported to reflect the activity and extent of UC [25], elevated titres in tolerant patients may represent inflammation rather than drug-specific intolerance. Thus, interpretation requires correlation with disease activity, endoscopic findings, and response to drug withdrawal or rechallenge. This study did not include a separate control group, which limits the interpretation of PR3-ANCA specificity for mesalazine intolerance. To set up the control group that does not receive mesalazine is difficult, because the drug is administered to nearly all patients diagnosed with UC. To further confirm the usefulness of serum PR3-ANCA levels in the diagnosis of mesalazine intolerance, multicenter prospective studies are required.

In conclusion, our study clearly demonstrated that serum PR3-ANCA levels were elevated in patients with UC and mesalazine intolerance. This marker could be useful for identifying mesalazine intolerance in patients in UC. Difficulty in discriminating mesalazine intolerance from UC exacerbation is a relevant and urgent problem in the clinical practice of UC. Although prospective studies with external validation are needed before routine clinical application, our results may help to resolve this problem and improve the disease course of patients with UC.

## Figures and Tables

**Table 1:** Comparison of the patient characteristics between the mesalazine intolerant and tolerant groups.

	Study Patients (*n* = 406)	Mesalazine Intolerant (*n* = 68)	Mesalazine Tolerant (*n* = 338)	*p*
Sex, male: female (male %)	225: 181 (55)	42: 26 (62)	183: 155 (54)	0.249
Age, years (IQR)	40 (26–54)	41 (25–55)	40 (26–54)	0.937
Age at diagnosis, years (IQR)	32 (21–46)	34 (20–52)	31 (21–46)	0.476
Disease duration, years (IQR)	2.5 (0.0–10.0)	1.0 (0.0–7.3)	3.0 (0.0–11.0)	0.027
Disease extent, n (%)				0.329
Extensive	227 (56)	41 (60)	186 (55)	
Left-sided	98 (24)	17 (25)	81 (24)	
Right-sided	30 (7)	6 (9)	24 (7)	
Rectum	51 (13)	4 (6)	47 (14)	
Extraintestinal manifestations, n (%)				
Primary sclerosing cholangitis	13 (3)	4 (6)	9 (3)	0.157
Arthritis	72 (18)	13 (19)	59 (17)	0.743
Skin lesions	24 (6)	5 (7)	19 (6)	0.374
Vasculitis	11 (3)	1 (1)	10 (3)	0.425
Eye lesions	4 (1)	1 (1)	3 (1)	0.521
Thrombosis	18 (4)	5 (7)	13 (4)	0.166
Medications, n (%)				
Mesalazine agents				
Oral treatments	272 (67)	30 (44)	242 (72)	<0.001
Topical treatments	42 (10)	4 (6)	38 (11)	0.185
Sulfasalazine	11 (3)	2 (3)	9 (3)	0.575
Corticosteroids				
Systemic treatments	87 (21)	21 (31)	66 (20)	0.037
Topical treatments	52 (13)	8 (12)	44 (13)	0.778
Thiopurines	56 (14)	12 (18)	44 (13)	0.312
Calcineurin inhibitors	11 (3)	4 (6)	7 (2)	0.094
Bio /JAK inhibitors	78 (19)	14 (21)	64 (19)	0.752
Others	3 (1)	0 (0)	3 (1)	0.576
pMayo score	2 (0–4)	2 (1–5)	2 (0–4)	0.156
CRP, mg/dL (IQR)	0.16 (0.04–1.17)	0.42 (0.08–5.03)	0.13 (0.03–0.87)	0.003
PR3-ANCA, U/mL (IQR)	1.9 (0.0–10.5)	4.5 (0.8–26.2)	1.5 (0.0–8.5)	0.001

Bio: biologics; CRP: C-reactive protein; IQR: interquartile range; JAK: Janus kinase; pMayo: partial Mayo; PR3-ANCA: proteinase 3 antineutrophil cytoplasmic antibody.

**Table 2:** Timings of PR3-ANCA measurement and the appearance of mesalazine intolerance.

Timings of the Measurement		PR3-ANCA (U/mL) Median (95%CI)
Before appearance	(n = 14)	4.6 (2.1–66.1)
Present	(n = 19)	17.3 (12.0–60.1)
<1 year after appearance	(n = 12)	3.0 (0.7–12.0)
>1 year after appearance	(n = 23)	1.8 (0.0–32.7)

PR3-ANCA: proteinase 3 antineutrophil cytoplasmic antibody.

**Table 3:** Analysis of the factors predicting mesalazine intolerance.

	Univariate Analysis OR (95% CI) *p*		Multivariate Analysis OR (95% CI) *p*	
Sex (male)	1.53 (0.47–5.01)	0.480		
Age (>40 years)	0.89 (0.31–2.51)	0.819		
Age at diagnosis (>32 years)	1.46 (0.52–4.15)	0.474		
Disease duration (≤2.5 years)	1.40 (0.46–4.24)	0.555		
Disease extent (Extensive)	1.28 (0.42–3.88)	0.668		
Any extraintestinal manifestations	0.80 (0.22–3.00)	0.745		
Medication				
Systemic corticosteroids	3.85 (1.31–11.28)	0.014	3.74 (1.14–12.33)	0.030
Topical corticosteroids	0.92 (0.24–3.43)	0.895		
Thiopurines	0.48 (0.06–3.86)	0.489		
Calcineurin inhibitors	0.00 (0.00)	0.999		
Bio/JAK inhibitors	0.72 (0.15–3.39)	0.678		
CRP (>0.16 mg/dL)	8.17 (1.05–63.69)	0.045	6.67 (0.79–56.50)	0.082
PR3-ANCA (>15.0 U/mL)	7.24 (2.42–21.72)	<0.001	8.25 (2.52–27.02)	<0.001

Bio: biologics; CI: confidence interval; CRP: C-reactive protein; JAK: Janus kinase; OR: odds ratio; PR3-ANCA: proteinase 3 antineutrophil cytoplasmic antibody.

## Data Availability

The datasets generated and/or analyzed during the current study are not publicly available considering that we did not receive the permission that data is currently accessible to researchers from outside organizations at present. However, it will be available from the corresponding author on reasonable request after permission will be obtained by the ethnic committee of the institution.

## Abbreviations

The following abbreviations are used in this manuscript:

ANCA	Antineutrophil cytoplasmic antibody
AUC	Area under the curve
Bio	Biologics
CI	Confidence interval
CRP	C-reactive protein
DLST	Drug-induced lymphocyte stimulation test
IQR	Interquartile range
JAK	Janus kinase
NET	Neutrophil extracellular trap
NF-κB	Nuclear factor-κB
OR	Odds ratio
pMayo	partial Mayo
PR3-ANCA	Proteinase 3 antineutrophil cytoplasmic antibody
ROC	Receiver operating characteristic
UC	Ulcerative colitis
